# An Analysis of Risk Factors for Radiation Necrosis Following Cranial Radiation

**DOI:** 10.7759/cureus.29268

**Published:** 2022-09-17

**Authors:** Caleb Nissen, Jun Ying, Madison Newkirk, Ganesh Narayanasamy, Gary Lewis, Fen Xia

**Affiliations:** 1 Radiation Oncology, Winthrop P. Rockefeller Cancer Institute, University of Arkansas for Medical Sciences, Little Rock, USA; 2 Biostatistics, Winthrop P. Rockefeller Cancer Institute, University of Arkansas for Medical Sciences, Little Rock, USA

**Keywords:** toxicity, radiation-induced brain necrosis, cranial radiation, radiation therapy, cerebral radiation necrosis

## Abstract

Introduction

Radiation necrosis in the brain is a frequent complication of brain radiation therapy (RT) and is characterized by various neurological symptoms including cognitive dysfunction, headaches, weakness, apraxia, aphasia, and numbness. These symptoms may be progressive and treatment-resistant. Currently, risk factors for radiation necrosis are not well characterized. The goal of this study is to identify risk factors for cerebral radiation necrosis in order to improve clinicians’ ability to appropriately weigh the risks and benefits of brain RT.

Methods

A retrospective chart review was performed on patients who were diagnosed with brain tumors and received RT (3D conformal therapy, volumetric modulated arc therapy, stereotactic radiosurgery, or stereotactic radiotherapy) at the University of Arkansas for Medical Sciences from July 1, 2017, to July 1, 2019. Data regarding demographics, characteristics of cancer, chemotherapy status and class, comorbidities, and additional medications of patients were collected via EPIC. Total RT dose, fraction size, volume of brain receiving 12 Gy (V12), and retreatment of locally recurrent tumors were recorded from Eclipse. The diagnosis of radiation necrosis was based on MRI reports that were examined for a time period of 24 months following the completion of radiation treatment and confirmed, when possible, by biopsy. Cases that did not have an MRI available at least two months after the completion of RT were excluded. Statistical association analyses were used to identify candidate risk factors to radiation necrosis. These candidate risk factors were further used to assess their associations to demographics and other characteristics of cancer and treatments. Finally, adjusted and unadjusted logistic regression models were used to predict radiation necrosis using a single risk factor or multiple risk factors. ROC curves were used to evaluate the performance of prediction or discrimination of the logistic regression models.

Results

A total of 139 patients were studied. The mean ± standard deviation (SD) for age was 60.4 ± 13.6 years, female:male ratio was 71:68, and White:African American:other race ratio was 112:24:3. A total of 43 (30.9%) patients were diagnosed with radiation necrosis. Radiation adjuvant to surgery, concurrent systemic therapy status, total dose, and V12 were found to be significantly associated with radiation necrosis and considered candidate risk factors of radiation necrosis in the study. Predictive models showed adjusted odds ratios ([aORs] 95% confidence intervals or CIs) of 3.70 (1.01-13.56) and 8.19 (1.78-37.78) with radiation adjuvant to surgery and concurrent systemic therapy, respectively. For every one unit (log-transformed) increase of total dose and V12, the aORs (95% CI’s) were 27.35 (3.74-200.16) and 1.63 (1.15-2.32), respectively.

Conclusion

Our study suggested a positive correlation of concurrent systemic therapy status and post-surgical adjuvant RT with the incidence of radiation necrosis. It further demonstrated that greater total RT dose and V12 were related to the risk of developing radiation necrosis following brain RT. Given the findings of this study, the aforementioned factors should be considered when weighing the risk of radiation necrosis with the benefits of treatment.

## Introduction

Radiation therapy (RT) has been a mainstay of treatment in the field of oncology, both as definitive and palliative treatment of benign and malignant tumors. It is not without side effects, as treatment-induced intracranial radiation necrosis (RN) represents a significant complication of brain irradiation resulting in a myriad of neurological symptoms including headaches, seizures, focal neurological deficits, and cognitive dysfunction [1]. The exact incidence of RN is difficult to ascertain due to variability in treatment modality, dose, and diagnostic criteria, but it has been shown to range from 5% to 50% [1]. Time available for follow-up further complicates estimations of RN incidence, as it frequently occurs between three months and several years post-RT [1]. Diagnosis can be made by MRI; however, RN frequently mimics tumor progression, and therefore a confirmatory biopsy is often needed.

Little is known about risk factors that may predispose to the development of NR. Tumor volume, number of isocenters, and total brain volume receiving a minimum of 8 to 16 Gy of radiation (V8-16) have been associated with RN in multiple studies [2-6]. Many other risk factors have been suggested; however, variability of findings across studies has been observed. The identification of risk factors and definition of the underlying mechanisms for brain NR will aid physicians in the assessment of individual patients and the planning of cranial radiation treatment to avoid/reduce NR. The goal of this study is to elucidate risk factors for NR in order to improve clinicians’ ability to appropriately weigh the risks and benefits of brain RT.

This article was previously presented as a meeting abstract at the 2022 ACRO Annual Meeting on March 11 and at the 2022 UAMS Cancer Intsitute retreat on May 26.

## Materials and methods

Data collection

A retrospective chart review was performed of patients who were diagnosed with primary or metastatic brain tumors and received RT (3D conformal therapy, volumetric modulated arc therapy, stereotactic radiosurgery, or stereotactic radiotherapy) at the University of Arkansas for Medical Sciences (UAMS) from July 1, 2017, to July 1, 2019. Patient demographics were collected from the electronic medical record. Medications including antidepressants (Y/N concurrent) and corticosteroids (Y/N one month before RT, during RT, or one month after RT) were determined from the patient medication history and radiation oncology treatment record. Exact corticosteroid doses were unable to be obtained as the taper instructions were rarely available. Systemic therapy status (six months before, during RT, or six months after RT) and systemic therapy class (immunotherapy, cytotoxic chemotherapy, targeted therapy) were determined in the same manner. Comorbidities (cardiovascular disease, hypertension, autoimmune, diabetes, psychiatric) were identified via the patient problem list. RT was considered to be adjuvant to surgery if RT to the surgical resection site occurred within six weeks of surgery according to surgical history and oncology treatment notes. Total RT dose, fraction size, fraction number, location (parietal, frontal, temporal, occipital, cerebellum, or brainstem) and retreatment of locally recurrent tumors were recorded from the treatment summary tab in Eclipse. Volume of normal brain tissue receiving 12 Gy (V12) was calculated using treatment planning in Eclipse for single and multifraction treatments, a radiation treatment planning software program. V12 was also converted to a biological equivalent dose at one fraction (V12 BED) for multifraction treatments by applying the linear-quadratic model (α/β = 10 for tumor; α/β = 3 for organs at risk. The diagnosis of NR (either symptomatic or asymptomatic) was based on MRI reports that were examined for a time period of 24 months following the completion of radiation treatment and confirmed, when possible, by biopsy. MRI reports that indicated a diagnosis or suspicion of NR were considered positive for NR. Symptomatic NR was defined by the presence of neurologic symptoms at the time of diagnosis according to clinic notes. Additionally, increased edema in the post-RT time period was also considered to be resultant of NR. Cases that did not have an MRI available at least two months after the completion of RT were excluded as the majority of RN takes time to develop.

Statistical analysis

All numerical variables were summarized in mean ± standard deviation (SD), and all categorical or binary variables were summarized in frequency (%) or odds. In the association analysis, t-tests were used to compare means of numerical variables between patients with NR and those without, and Pearson’s chi-square tests were used to compare frequencies or odds between groups for categorical or binary variables. Variables showing significant associations to NR were considered candidate risk factors of NR. They were further assessed of their associations to demographics and other cancer-related characteristics using ANOVA models and Pearson’s chi-square tests, depending on if they were numerical or categorical risk factors. Finally, in the predictive analysis, candidate risk factors were used to predict NR using logistical regression models, using both unadjusted and adjusted approaches. The unadjusted approach used one risk factor as the single predictor or multiple risk factors as predictors in the models, while the adjusted approach included demographics and other cancer-related characteristics as additional independent variables in the models. From each of the logistic regression models, a receiver operating characteristic (ROC) curve was used to assess the performance of prediction or discrimination. Specifically, an area under the ROC curve (AUC) was used to assess the overall performance of prediction or discrimination. At each point of the ROC curve, its corresponding sensitivity and specificity were also calculated. The optimal point at which the sum of sensitivity and specificity was the largest was identified and used to represent the best cut off threshold of the logit score in predicting or discriminating NR using a candidate risk factor or risk factors. The AUC (or sensitivity or specificity) was considered outstanding, excellent, good, fair, or poor if its value was in the range of 0.9-1.0, 0.81-0.90, 0.71-0.80, 0.61-0.70, and <0.60, respectively. All statistical models and tests were computed using SAS 9.4 software (SAS Institute Inc., Cary, NC). P-values < 0.05 were considered statistically significant. Numerical variables were inspected of empirical distributions before formal analysis to ensure that they met the conditions for parametric statistical models. Transformed variables were used otherwise.

## Results

A total of 139 patients were studied. Mean ± SD for age was 60.4 ± 13.6 years, female:male ratio was 71:68, and White:African American:other race ratio was 112:24:3. Data on patient demographics, tumor type, and radiotherapy characteristics are shown in Table 1. A total of 43 (30.9%) patients were diagnosed by MRI with NR. Overall, 13.4% of cases had biopsy results available, of which 100% concurred with the radiologist's diagnosis of NR, and 53.5% (23/43) of patients with NR were symptomatic at the time of diagnosis by MRI. The fractionations and total doses of symptomatic and asymptomatic NR are shown in Table 2.

**Table 1:** Patient demographics, tumor types, and radiotherapy characteristics *Some patients received multiple treatments to different sites with varying fraction numbers

Patient and treatment characteristics	Number	%
Gender		
Female	71	51.1
Male	68	48.9
Race		
White	112	80.6
African American	24	17.3
Other	3	2.1
Tumor type		
Primary		
Meningioma	23	16.5
Malignant glioma	19	13.7
Hemangioblastoma	1	0.7
Neurocytoma	1	0.7
Medulloblastoma	1	0.7
Pituitary microadenoma	1	0.7
Pinealblastoma	1	0.7
Metastatic (histology and original site)		
Adenocarcinoma (lung)	31	22.3
Squamous cell carcinoma (lung)	8	5.8
Non-small cell lung cancer, not otherwise specified	6	4.3
Small-cell lung cancer	5	3.6
Melanoma	13	9.4
Renal cell carcinoma	10	7.2
Invasive ductal carcinoma (breast)	9	6.5
Adenocarcinoma (colorectal)	4	2.9
Papillary carcinoma (thyroid)	2	1.4
Adenocarcinoma (sinus)	1	0.7
Urothelial carcinoma (bladder)	1	0.7
Serous carcinoma (ovary)	1	0.7
Adenocarcinoma (prostate)	1	0.7
Treatment fractions and dose range*		
1 fraction, 13-24 Gy	106	69.3
2 fractions, 18-20 Gy	2	1.3
3 fractions, 21-30 Gy	10	6.5
5 fractions, 20-35 Gy	15	9.8
10 fractions, 30-40 Gy	3	2
20 fractions, 40 Gy	1	0.7
25 fractions, 50 Gy	1	0.7
28 fractions, 50.4 Gy	1	0.7
29 fractions, 58-59.45 Gy	5	3.3
30 fractions, 60 Gy	9	5.9

**Table 2:** Radiotherapy treatment characteristics of patients who developed symptomatic and asymptomatic radiation necrosis RN, radiation necrosis

Treatment fractions and dose	Number	%
Symptomatic RN		
1 fraction, 15 Gy-20 Gy	15	68.2
5 fractions, 35 Gy	1	4.5
29 fractions, 59 Gy	2	9.1
30 fractions, 60 Gy	4	18.2
Asymptomatic RN		
1 fraction, 15-20 Gy	12	57.1
3 fractions, 30 gy	1	4.8
5 fractions, 35 Gy	3	14.3
20 fractions, 40 Gy	1	4.8
29 fractions, 59 Gy	1	4.8
30 fractions, 60 Gy	3	14.3

Total dose of RT and V12 were found to be higher in patients with necrosis (mean ± SD = 7.90 ± 0.49 and 2.56 ± 1.73, respectively) than those without necrosis (mean ± SD = 7.66 ± 0.31 and 3.54 ± 2.37, p-values < 0.01). V12 biologic equivalent dose at one fraction was also shown to be higher in patients with necrosis than those without necrosis (mean ± SD = 3.21 ± 2.03 vs. 2.30 ± 1.48, p-value = 0.0035). Additionally, patients with necrosis were more likely to have concurrent systemic therapy status and RT adjuvant to surgery than those without necrosis, with odds ratios being 3.23 and 2.29 (p-values = 0.021 and 0.032), respectively. No other associations were identified. The association analysis in its entirety is shown in Tables 3, 4.

**Table 3:** Comparisons of frequency of tumor and treatment-related characteristics between patients with necrosis and with no necrosis. Re-irradiation: RT applied to a previously treated contour. Additional RT: RT to this target that was not accounted for by another case in this study (whole brain RT or RT which occurred after July 1, 2019, but while follow-up was still occurring) *N=61 under no radiation necrosis and N=33 under radiation necrosis. RT, radiation therapy

Measure	Definition	No radiation necrosis (N=96)	Radiation necrosis (N=43)	p-Value
Medications	Corticosteroids	83 (86.5%)	39 (90.7%)	0.4807
	Antidepressants	29 (30.2%)	12 (27.9%)	0.7833
Comorbidities	Diabetes	14 (14.6%)	3 (7.0%)	0.2058
	Autoimmune disease	2 (2.1%)	0 (0.0%)	0.3404
	Hypertension	34 (35.4%)	15 (34.9%)	0.9515
	Cardiovascular disease	25 (26.0%)	6 (14.0%)	0.1135
	Psychiatric disorder	16 (16.7%)	3 (7.0%)	0.1242
Cancer location	Brainstem	7 (7.3%)	3 (7.0%)	0.9470
	Cerebellum	28 (29.2%)	8 (18.6%)	0.1889
	Frontal	55 (57.3%)	23 (53.5%)	0.6762
	Occipital	23 (24.0%)	14 (32.6%)	0.2890
	Parietal	27 (28.1%)	16 (37.2%)	0.2842
	Temporal	17 (17.7%)	13 (30.2%)	0.0971
Systemic chemotherapy status*	Concurrent	30 (49.2%)	25 (73.5%)	0.0212
Systemic chemotherapy class*	Cytotoxic	26 (42.6%)	16 (48.5%)	0.5853
	Targeted	19 (31.1%)	6 (18.2%)	0.1745
	Immunotherapy	27 (44.3%)	13 (39.4%)	0.6486
	Not specified	2 (3.3%)	3 (9.1%)	0.2307
RT adjuvant to surgery	Yes	23 (24.0%)	18 (41.9%)	0.0324
Number of fractions	≥10	9 (9.4%)	12 (27.9%)	0.0048
Re-irradiation	Yes	16 (16.7%)	10 (23.3%)	0.3571
Additional RT	Yes	1 (1.0%)	4 (9.3%)	0.0156
Primary brain tumor	Yes	31 (32.3%)	16 (37.2%)	0.5711
Tumor counts	>4	12 (12.5%)	11 (25.6%)	0.0551

**Table 4:** Comparisons of total dose, fraction size, and volume between patients with necrosis and with no necrosis. BED, biologic equivalent dose; V12, volume of brain tissue receiving 12-Gy dose

Variable	No radiation necrosis (N=96)	Radiation necrosis (N=43)	p-Value
Total dose (Gy)	7.66 ± 0.31	7.90 ± 0.49	0.0007
Fraction size (Gy)	2.27 ± 0.56	2.07 ± 0.72	0.0771
V12 (cc/cm)	2.56 ± 1.73	3.54 ± 2.37	0.0070
V12 BED equivalent at one fraction (cc/cm)	2.30 ± 1.48	3.21 ± 2.03	0.0035

Covariate analysis was performed analyzing the associations of candidate risk factors versus other characteristics (demographics, cancer treatment, etc.) and is summarized in Tables 5, 6.

**Table 5:** Covariate analysis of total dose and volume vs. demographic and medical characteristics. BED, biologic equivalent dose; V12, volume of brain tissue receiving 12-Gy dose

Covariate	Group	Total dose (mean ± SD)	V12 BED equivalent at one fraction (mean ± SD)
Medication			
Corticosteroids	No	7.82 ± 0.44	2.82 ± 1.88
	Yes	7.72 ± 0.38	2.55 ± 1.69
	p-value	0.3180	0.5513
Antidepressants	No	7.72 ± 0.38	2.60 ± 1.69
	Yes	7.77 ± 0.41	2.56 ± 1.78
	p-value	0.4499	0.8996
Comorbidity			
Diabetes	No	7.75 ± 0.41	2.65 ± 1.76
	Yes	7.61 ± 0.18	2.12 ± 1.30
	p-value	0.1582	0.2334
Autoimmune disease	No	7.74 ± 0.39	2.58 ± 1.73
	Yes	7.56 ± 0.13	2.74 ± 0.07
	p-value	0.5263	0.8971
Hypertension	No	7.76 ± 0.44	2.77 ± 1.85
	Yes	7.68 ± 0.28	2.25 ± 1.38
	p-value	0.2598	0.0878
Cardiovascular disease	No	7.75 ± 0.42	2.67 ± 1.74
	Yes	7.68 ± 0.28	2.29 ± 1.62
	p-value	0.3635	0.2747
Psychiatric disorder	No	7.74 ± 0.39	2.63 ± 1.71
	Yes	7.70 ± 0.37	2.28 ± 1.73
	p-value	0.6727	0.4017
Cancer location			
Brainstem	No	7.75 ± 0.40	2.70 ± 1.68
	Yes	7.57 ± 0.19	1.12 ± 1.50
	p-value	0.1778	0.0046
Cerebellum	No	7.77 ± 0.42	2.73 ± 1.83
	Yes	7.63 ± 0.25	2.18 ± 1.25
	p-value	0.0635	0.0974
Frontal	No	7.81 ± 0.47	2.87 ± 1.86
	Yes	7.67 ± 0.30	2.36 ± 1.57
	p-value	0.0357	0.0832
Occipital	No	7.78 ± 0.43	2.65 ± 1.81
	Yes	7.61 ± 0.18	2.41 ± 1.43
	p-value	0.0217	0.4707
Parietal	No	7.76 ± 0.43	2.68 ± 1.80
	Yes	7.68 ± 0.26	2.36 ± 1.49
	p-value	0.3004	0.3119
Temporal	No	7.75 ± 0.41	2.66 ± 1.71
	Yes	7.69 ± 0.32	2.31 ± 1.72
	p-value	0.5041	0.3224
Systemic			
Cytotoxic	No	7.62 ± 0.15	2.01 ± 1.24
	Yes	8.01 ± 0.52	3.48 ± 2.19
	p-value	0.0000	0.0001
Targeted	No	7.85 ± 0.45	2.77 ± 2.09
	Yes	7.64 ± 0.20	2.38 ± 1.07
	p-value	0.0260	0.3798
Immunotherapy	No	7.91 ± 0.50	3.20 ± 2.08
	Yes	7.64 ± 0.14	1.96 ± 1.26
	p-value	0.0014	0.0012
On chemo but not specified	No	7.81 ± 0.42	2.75 ± 1.89
	Yes	7.58 ± 0.03	1.29 ± 0.85
	p-value	0.2297	0.0912
Retreatment	No	7.77 ± 0.42	2.72 ± 1.76
	Yes	7.60 ± 0.11	1.99 ± 1.39
	p-value	0.0428	0.0513
Primary tumor	No	7.62 ± 0.14	2.08 ± 1.24
	Yes	7.97 ± 0.58	3.56 ± 2.07
	p-value	0.0000	0.0000
Age>60	No	7.83 ± 0.45	2.79 ± 1.84
	Yes	7.65 ± 0.30	2.39 ± 1.58
	p-value	0.0056	0.1661
Male	No	7.69 ± 0.37	2.39 ± 1.62
	Yes	7.78 ± 0.40	2.79 ± 1.80
	p-value	0.2093	0.1663
White	No	7.72 ± 0.35	2.71 ± 1.76
	Yes	7.74 ± 0.40	2.55 ± 1.71
	p-value	0.8732	0.6765

**Table 6:** Covariate analysis of risk factors vs. demographic and medical characteristics. Re-irradiation, RT applied to a previously treated contour; additional RT, RT to this target that was not accounted for by another case in this study (whole brain RT or RT which occurred after July 1, 2019, but while follow-up was still occurring) RT, radiation therapy

Covariate	Group	RT adjuvant to surgery	Concurrent systemic chemotherapy	Number of fractions ≥ 10	Additional RT
Medication					
Corticosteroids	No	3/17 (17.6%)	3/8 (37.5%)	4/17 (23.5%)	0/17 (0.0%)
	Yes	38/122 (31.1%)	34/87 (39.1%)	17/122 (13.9%)	5/122 (4.1%)
	p-value	0.2528	0.9301	0.3007	0.3953
Antidepressants	No	32/98 (32.7%)	26/67 (38.8%)	14/98 (14.3%)	3/98 (3.1%)
	Yes	9/41 (22.0%)	11/28 (39.3%)	7/41 (17.1%)	2/41 (4.9%)
	p-value	0.2070	0.9651	0.6756	0.5999
Comorbidity					
Diabetes	No	36/122 (29.5%)	30/82 (36.6%)	21/122 (17.2%)	4/122 (3.3%)
	Yes	5/17 (29.4%)	7/13 (53.8%)	0/17 (0.0%)	1/17 (5.9%)
	p-value	0.9935	0.2357	0.0634	0.5891
Autoimmune disease	No	40/137 (29.2%)	37/93 (39.8%)	21/137 (15.3%)	5/137 (3.6%)
	Yes	1/2 (50.0%)	0/2 (0.0%)	0/2 (0.0%)	0/2 (0.0%)
	p-value	0.5219	0.2536	0.5479	0.7832
Hypertension	No	25/90 (27.8%)	24/59 (40.7%)	17/90 (18.9%)	2/90 (2.2%)
	Yes	16/49 (32.7%)	13/36 (36.1%)	4/49 (8.2%)	3/49 (6.1%)
	p-value	0.5471	0.6579	0.0916	0.2381
Cardiovascular disease	No	31/108 (28.7%)	26/74 (35.1%)	19/108 (17.6%)	5/108 (4.6%)
	Yes	10/31 (32.3%)	11/21 (52.4%)	2/31 (6.5%)	0/31 (0.0%)
	p-value	0.7021	0.1526	0.1268	0.2224
Psychiatric disorder	No	36/120 (30.0%)	30/82 (36.6%)	19/120 (15.8%)	5/120 (4.2%)
	Yes	5/19 (26.3%)	7/13 (53.8%)	2/19 (10.5%)	0/19 (0.0%)
	p-value	0.7435	0.2357	0.5484	0.3648
Cancer location					
Brainstem	No	41/129 (31.8%)	33/89 (37.1%)	21/129 (16.3%)	5/129 (3.9%)
	Yes	0/10 (0.0%)	4/6 (66.7%)	0/10 (0.0%)	0/10 (0.0%)
	p-value	0.0337	0.1503	0.1661	0.5260
Cerebellum	No	30/103 (29.1%)	21/65 (32.3%)	18/103 (17.5%)	2/103 (1.9%)
	Yes	11/36 (30.6%)	16/30 (53.3%)	3/36 (8.3%)	3/36 (8.3%)
	p-value	0.8714	0.0508	0.1873	0.0763
Frontal	No	18/61 (29.5%)	16/38 (42.1%)	13/61 (21.3%)	1/61 (1.6%)
	Yes	23/78 (29.5%)	21/57 (36.8%)	8/78 (10.3%)	4/78 (5.1%)
	p-value	0.9978	0.6063	0.0709	0.2730
Occipital	No	27/102 (26.5%)	25/68 (36.8%)	20/102 (19.6%)	2/102 (2.0%)
	Yes	14/37 (37.8%)	12/27 (44.4%)	1/37 (2.7%)	3/37 (8.1%)
	p-value	0.1940	0.4887	0.0139	0.0854
Parietal	No	34/96 (35.4%)	27/61 (44.3%)	17/96 (17.7%)	3/96 (3.1%)
	Yes	7/43 (16.3%)	10/34 (29.4%)	4/43 (9.3%)	2/43 (4.7%)
	p-value	0.0222	0.1547	0.2008	0.6551
Temporal	No	33/109 (30.3%)	28/73 (38.4%)	16/109 (14.7%)	3/109 (2.8%)
	Yes	8/30 (26.7%)	9/22 (40.9%)	5/30 (16.7%)	2/30 (6.7%)
	p-value	0.7011	0.8296	0.7878	0.3079
Systemic chemotherapy					
Cytotoxic	No	13/52 (25.0%)	19/52 (36.5%)	0/52 (0.0%)	4/52 (7.7%)
	Yes	18/42 (42.9%)	17/42 (40.5%)	18/42 (42.9%)	1/42 (2.4%)
	p-value	0.0671	0.6962	0.0000	0.2539
Targeted	No	25/69 (36.2%)	29/69 (42.0%)	17/69 (24.6%)	5/69 (7.2%)
	Yes	6/25 (24.0%)	7/25 (28.0%)	1/25 (4.0%)	0/25 (0.0%)
	p-value	0.2650	0.2164	0.0246	0.1666
Immunotherapy	No	21/54 (38.9%)	14/54 (25.9%)	17/54 (31.5%)	2/54 (3.7%)
	Yes	10/40 (25.0%)	22/40 (55.0%)	1/40 (2.5%)	3/40 (7.5%)
	p-value	0.1567	0.0041	0.0004	0.4174
Not specified	No	30/89 (33.7%)	34/89 (38.2%)	18/89 (20.2%)	4/89 (4.5%)
	Yes	1/5 (20.0%)	2/5 (40.0%)	0/5 (0.0%)	1/5 (20.0%)
	p-value	0.5258	0.9359	0.2634	0.1328
Re-irradiation	No	33/113 (29.2%)	28/75 (37.3%)	19/113 (16.8%)	3/113 (2.7%)
	Yes	8/26 (30.8%)	9/20 (45.0%)	2/26 (7.7%)	2/26 (7.7%)
	p-value	0.8746	0.5321	0.2416	0.2136
Primary tumor	No	25/92 (27.2%)	36/77 (46.8%)	2/92 (2.2%)	5/92 (5.4%)
	Yes	16/47 (34.0%)	1/18 (5.6%)	19/47 (40.4%)	0/47 (0.0%)
	p-value	0.4009	0.0013	0.0000	0.1036
Age > 60	No	24/67 (35.8%)	17/51 (33.3%)	16/67 (23.9%)	2/67 (3.0%)
	Yes	17/72 (23.6%)	20/44 (45.5%)	5/72 (6.9%)	3/72 (4.2%)
	p-value	0.1147	0.2270	0.0053	0.7086
Male	No	21/71 (29.6%)	16/44 (36.4%)	9/71 (12.7%)	3/71 (4.2%)
	Yes	20/68 (29.4%)	21/51 (41.2%)	12/68 (17.6%)	2/68 (2.9%)
	p-value	0.9829	0.6314	0.4133	0.6844
White	No	7/27 (25.9%)	5/19 (26.3%)	5/27 (18.5%)	1/27 (3.7%)
	Yes	34/112 (30.4%)	32/76 (42.1%)	16/112 (14.3%)	4/112 (3.6%)
	p-value	0.6504	0.2068	0.5814	0.9736

**Figure 1:**
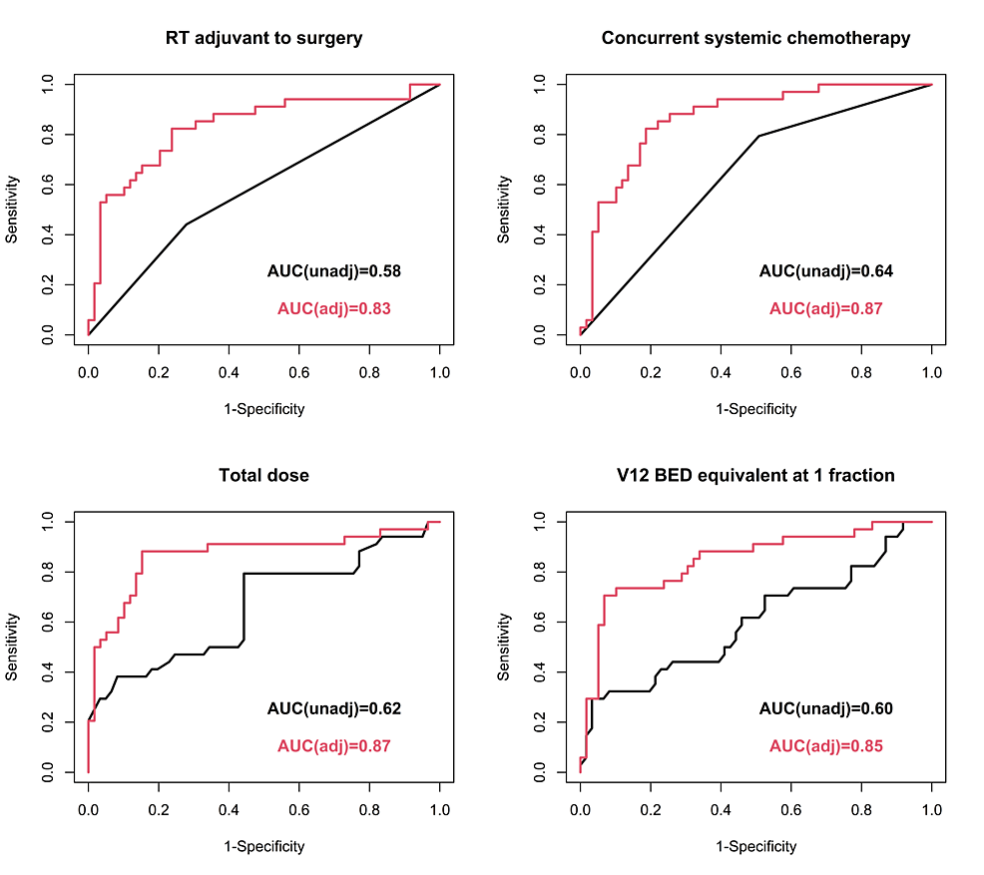
ROC curves using adjusted and unadjusted logistic regression models AUC, area under curve; BED, biologic equivalent; ROC, receiver operating characteristic; RT, radiation therapy; V12, volume of brain tissue receiving 12-Gy dose

The unadjusted odds ratio for total dose and V12 BED were 4.63 and 1.30 (90% CI = 1.57-13.67 and 1.02-1.64; p = 0.005 and 0.038), respectively, and the adjusted odds ratio were 27.35 and 1.74 (90% CI = 3.74-200.16 and 1.17-2.58; p = 0.001 and 0.006), respectively. Concurrent systemic therapy status also increased the risk of developing RN with an unadjusted odds ratio of 3.73 and an adjusted odds ratio of 8.19 (90% CI = 1.41-9.88 and 1.78-37.78, respectively). RT adjuvant to surgery did not increase the risk of RN in the unadjusted model with an odds ratio of 2.04 (90% CI = 0.85-4.93, p = 0.111). However, the adjusted model exhibited an odds ratio of 3.70 (90% CI = 1.01-13.56, p = 0.048). No statistical correlation was observed between specific systemic therapy class (immunotherapy, cytotoxic chemotherapy, targeted therapy) and incidence of RN. Number of fractions ≥ 10 also increased the risk of RN with an unadjusted odds ratio of 5.00 and an adjusted odds ratio of 20.15 (90% CI = 1.66-15.03 and 3.03-134.11; p = 0.004 and 0.002). Predictive model results are found in their entirety in Tables 7-10. Figure 1 shows ROC curves. Demographics, tumor location, corticosteroids, antidepressants, and comorbidities (cardiovascular disease, hypertension, diabetes, autoimmune, psychiatric) further did not have any statistically significant effect on the development of brain RN.

**Table 7:** Prediction of cerebral radiation necrosis using unadjusted risk factors. BED, biologic equivalent; RT, radiation therapy; V12, volume of brain tissue receiving 12-Gy dose

Risk factor	OR	95% CI	p-Value
RT adjuvant to surgery	2.04	(0.85-4.93)	0.111
Concurrent systemic chemotherapy	3.73	(1.41-9.88)	0.008
Total dose	4.63	(1.57-13.67)	0.005
V12 BED equivalent at one fraction	1.30	(1.02-1.64)	0.031

**Table 8:** Prediction of cerebral radiation necrosis using adjusted risk factors. BED, biologic equivalent; RT, radiation therapy; V12, volume of brain tissue receiving 12-Gy dose

Risk factor	Odds ratio	95% CI	p-Value
RT adjuvant to surgery	3.70	(1.01-13.56)	0.048
Concurrent systemic chemotherapy	8.19	(1.78-37.78)	0.007
Total Dose	27.35	(3.74-200.16)	0.001
V12 BED equivalent at 1 fraction	1.74	(1.17-2.58)	0.006

**Table 9:** Summary of AUC, sensitivity, and specificity from ROC curves using unadjusted logistic regression models. AUC, area under curve; BED, biologic equivalent; ROC, receiver operating characteristic; RT, radiation therapy; V12, volume of brain tissue receiving 12-Gy dose

Risk factor	AUC	Sensitivity	Specificity
RT adjuvant to surgery	58.12%	44.12%	72.13%
Concurrent systemic chemotherapy	64.30%	79.41%	49.18%
Total dose	61.86%	38.24%	90.16%
V12 BED equivalent at one fraction	59.88%	32.35%	90.16%

**Table 10:** Summary of AUC, sensitivity, and specificity from ROC curves using adjusted logistic regression models. AUC, area under curve; BED, biologic equivalent; ROC, receiver operating characteristic; RT, radiation therapy; V12, volume of brain tissue receiving 12-Gy dose

Risk factor	AUC	Sensitivity	Specificity
RT adjuvant to surgery	83.35%	82.35%	76.27%
Concurrent systemic chemotherapy	86.64%	82.35%	81.36%
Total dose	86.84%	88.24%	84.75%
V12 BED equivalent at one fraction	84.75%	70.59%	93.22%

## Discussion

The 30.9% incidence of RN observed in this study is comparable with those measured by previous studies, although a wide range of 5-50% has been reported in the literature. This is likely due to variations in treatment modality, dose, and diagnostic criteria [1]. Our study diagnosed RN based on MRI findings that were confirmed, when possible, with brain biopsy. Accuracy of RN diagnosis is limited by the availability of confirmatory biopsies as RN frequently mimic tumor progression. Confirmatory biopsies were available 13.4% of the time. A certain level of ambiguity is to be expected in any RN study, as performing brain biopsies on every patient with suspicion for RN would be poor practice.

We found V12, V12 BED, and total radiation dose to be associated with an increased risk of developing RN. These findings were consistent with the majority of the literature on RN risk factors. Minniti et al. found V10-V16 to have the highest predictive value of any risk factor on multivariate analysis [3]. Blonigen et al. further demonstrated that V8-V16 is correlated with RN [4]. Milano et al. performed a literature review and found that V12 was predictive for brain metastases treated with single fraction SRS. Additionally, in brain metastases with V20 (3-fractions) or V24 (5-fractions) < 20 cm3, the risk of RN was reduced by 10% [7]. Nedzi et al. showed an association on univariate analysis between RN and maximum tumor dose and maximum normal tissue dose [2]. Zhuang et al. data suggested that biologically equivalent dose could predict RN [6]. Ruben et al. demonstrated total dose to be a significant risk factor of RN [8].

Additional findings include a positive correlation of NR with concurrent systemic therapy status. We hypothesized that immunotherapies specifically would have the greatest effect on RN incidence; however, when subcategorizing, no specific systemic therapy class was found to correlate with RN risk in our study. On covariate analysis, systemic cytotoxic chemotherapy was found to be correlated with increased total dose and V12. This is unexpected as patients on chemotherapy generally receive lower doses of radiation in clinical practice. These findings could be a result of including glioma patients in this analysis, as these patients receive higher doses of RT along with concurrent cytotoxic chemotherapy. As expected, targeted systemic therapy demonstrated an association with lower total doses of radiation for the management of metastatic tumor, and immunotherapy demonstrated an association with both lower total RT doses and lower V12. Furthermore, we believe the association shown on univariate analysis is legitimate as the varying associations between chemotherapy class and total dose and V12 balance out. This association with chemotherapy and brain RN was also demonstrated by Ruben et al. in patients undergoing treatment for glioma [8]. Kim et al. found that concurrent chemotherapy with VEGFR tyrosine kinase inhibitors and EGFR receptor tyrosine kinase inhibitors increased the risk of RN [9].

Our data demonstrated that patients with necrosis were more likely to have RT adjuvant to surgery than those without necrosis, with an odds ratio of 2.29 (p= 0.032). However, when using post-resection status to predict RN, the results were insignificant (OR: 2.04; 90% CI = 0.85-4.93; p = 0.111). When using post-resection status to predict RN as a part of an adjusted model considering comorbidities, the results were significant with an OR of 3.70 (90% CI = 1.01-13.56; p = 0.048). To our knowledge, the relationship between RT adjuvant to surgery and RN demonstrated in this study has yet to be demonstrated in the literature; however, this relationship makes sense on a physiological level. The increased risk of RN in patients receiving RT adjuvant to surgery is likely secondary to an increase in inflammation of the surgical area as an adverse side effect of surgical insults to brain tissue.

Our research also showed an increased risk of cerebral RN for patients receiving ≥10 fractions of RT mostly in cases of primary brain tumors. This is likely due to the increased total dose seen in these patients and not as a result of number of fractions by itself. Minniti et al. showed that multifraction RT results in lower rates of RN when compared to single fraction in metastatic brain disease [10].

Our study found no relationship between corticosteroids or antidepressants and RN. An effect of any immunologically active drug on RN would follow logically and physiologically; however, this study found no such correlation. One limitation here is the lack of information on steroid taper doses for patients on corticosteroids, as these data were found to be unavailable upon chart review. Thus, it is possible that higher doses of corticosteroids affect the risk of RN; however, this effect would be masked in our study by the presence of patients on lower dose tapers. To our knowledge, no other studies have examined the effect of concurrent corticosteroid or antidepressant treatment on the risk of developing brain RN.

Retreatment of locally recurring tumors, tumor location, and comorbidities (cardiovascular disease, hypertension, diabetes, autoimmune, psychiatric) did not demonstrate a statistically significant association with RN in our study. Minniti et al.’s data showed that parietal location was associated with brain RN [3]; however, other studies have not shown such an association. Sayan et al. found that diabetes was associated with the development of symptomatic brain RN for patients with brain metastases [11].

Our statistical analysis examined symptomatic and asymptomatic RN as a combined entity. We believe that this is not problematic for several reasons. For one, asymptomatic necrosis is still clinically significant as symptoms often develop long after it is diagnosed on MRI. There is also no consensus in how symptomatic NR is defined as the time to development of symptoms varies. Furthermore, our data demonstrated that similar radiotherapy treatment regimens were seen in symptomatic and asymptomatic RN. Finally, previous studies have also shown that both sympomatic and asymptomatic RN are associated with V12 which helps validate our results [3]. There is certainly value in examining these two entities separately, and future prospective research should be conducted in a larger scale study with this goal in mind.

## Conclusions

Our study suggested a positive correlation of concurrent systemic therapy status and post-surgical adjuvant RT with the incidence of NR. It further demonstrated that greater total RT dose, V12, and V12 BED are related to the risk for developing NR following brain RT. Given the findings of this study, the aforementioned factors should be considered when weighing the risk of NR with the benefits of treatment. Further studies should be conducted with emphasis placed on systemic therapy class, diabetic status, and concurrent corticosteroid dose.
